# Diurnal changes of noninvasive parameters of ocular surface in healthy subjects before and after continuous face mask wearing during the COVID-19 pandemic

**DOI:** 10.1038/s41598-022-17486-4

**Published:** 2022-07-29

**Authors:** Giuseppe Giannaccare, Marco Pellegrini, Massimiliano Borselli, Carlotta Senni, Angela Bruno, Vincenzo Scorcia

**Affiliations:** 1 Department of Ophthalmology, University “Magna Græcia” of Catanzaro, Viale Europa, 88100 Germaneto, Catanzaro Italy; 2 Department of Translational Medicine, University of Ferrara, Ferrara, Italy; 3 Department of Ophthalmology, Ospedali Privati Forlì “Villa Igea”, Forlì, Italy; 4 Istituto Internazionale per la Ricerca e Formazione in Oftalmologia, Forlì, Italy; 5 Department of Ophthalmology, University Vita-Salute-IRCCS Ospedale San Raffaele, Milan, Italy

**Keywords:** Health care, Medical research

## Abstract

To investigate whether diurnal changes in noninvasive ocular surface parameters and subjective symptoms occur in healthy subjects wearing face mask who were analyzed before and after 8 h of continuous use. In this prospective cross-sectional study, healthy volunteers attending the same workplace environment underwent a noninvasive ocular surface workup by means of Keratograph 5 M (Oculus, Wetzlar, Germany) in the same day at 2 different time points: (i) in the early morning before wearing face mask (T0); (ii) after 8 h of continuous face mask use (T1). Noninvasive break-up time (NIBUT), tear meniscus height (TMH), ocular redness and meibomian gland dropout were measured. All subjects were asked to complete the Ocular Surface Disease Index (OSDI) questionnaire before and after 8 h of face mask wearing. Data from 20 healthy subjects (10 males and 10 females, mean age 25.1 ± 3.9 years) were included. Mean value of TMH decreased significantly from 0.29 ± 0.07 at T0 to 0.23 ± 0.07 mm at T1 (*P* < 0.001); conversely, mean values of NIBUT, redness score and meibomian gland dropout did not change significantly after continuous face mask wearing (always *P* > 0.532). Concerning ocular discomfort symptoms, mean value of OSDI score worsened significantly at T1 compared to T0 (from 12.9 ± 12.6 to 19.4 ± 12.0; *P* = 0.017). Continuous face mask wearing for 8 h led to decreased TMH associated with the onset of ocular discomfort symptoms in young healthy subjects.

## Introduction

Because of the coronavirus disease 2019 (COVID-19) pandemic, important public health measures including the use of personal protective precautions have been imposed in order to decrease viral transmission. Among these, face mask wearing is one of the main measures that has become mandatory for all members of the society. In particular, the Italian government impose the use of FFP2 or KN95 masks to enter hospitals for both patients and healthcare professionals. Although effective in controlling the spread of viral infection^1,2^, prolonged face mask use has been associated with different ocular conditions, like infectious keratitis, recurrent chalazion and mask-associated dry eye (MADE)^3–12^. Moshirfar et al. first described this condition which is more pronounced in subjects with occupations mandating prolonged use of face-masks on a regular basis, such as healthcare personnel, as well as in patients with pre-existing dry eye disease^5^. The primary mechanism for the onset of MADE is the poor fitting of the upper mask edge, resulting in the leakage of air towards the ocular surface^6^. A similar mechanism has been previously described to a greater extent for the occurrence of dry eye and corneal damage in patients using continuous positive airway pressure devices to treat obstructive sleep apnea^13^.

Although there have been several reports describing ocular irritation and dryness associated with regular mask use^5–11^, to date there are no objective measurements demonstrating diurnal alterations of ocular surface parameters occurring in the same subjects before and after the use of face mask. The recent development of all-in-one imaging devices allows the automated, objective and noninvasive measurements of various parameters of the ocular surface with good values of reproducibility^13–15^.

This study was designed to investigate whether diurnal changes in noninvasive ocular surface parameters and subjective symptoms occur in healthy subjects wearing face mask who were analyzed before and after 8 h of continuous use.

## Methods

This prospective cross-sectional study was conducted at the Department of Ophthalmology of the University Hospital of Catanzaro (Italy) between December 2021 and February 2022. Healthy volunteers attending the same workplace environment (students in the Faculty of Medicine and Surgery or in the Course of Orthoptic and Ophthalmologic Assistance of the University Magna Graecia of Catanzaro) were recruited. Written informed consent was obtained from all the patients, and the study was approved by the local Ethics Committee (Comitato Etico Regione Calabria Sezione Area Centro). The study was performed in accordance with the tenets of the Declaration of Helsinki. Exclusion criteria were: age under 18 years, previous diagnosis of COVID-19 infection, history of allergy, ocular surgery or other ocular surface diseases such as dry eye, contact lens wearing and use of any topical medication including also tear substitutes.

All subjects underwent a noninvasive ocular surface workup by means of Keratograph 5 M (Oculus, Wetzlar, Germany) in the same day at 2 different time points: i) in the early morning before wearing face mask (at 8 am) (T0); ii) after 8 h of continuous face mask use (at 4 pm) (T1). Ocular surface workout included the measurement of the following parameters: noninvasive break-up time (NIBUT), tear meniscus height (TMH), ocular redness and infrared meibography of the inferior eye lid^13–15^. Meibomian gland dropout was then calculated by means of the image editing software ImageJ (National Institute of Health; http://imagej.nih.gov/ij) as the percentage of gland loss in relation to the total tarsal area of the lid^16,17^. All subjects were asked to complete the Ocular Surface Disease Index (OSDI) questionnaire providing the responses related to the condition of their eyes before and after 8 h of face mask wearing.

After the initial examination, all subjects were provided the same model of KN95 face mask, equipped with a built-in metallic nasal strip to affix mask across the nasal bridge (Suvayu SV9500, Aamr Lifesciences, India). Face mask airtightness testing was conducted in 3 steps, as recently described^18^: first, face masks were observed to identify potential gaps between face mask edges and participant’s face; second, cotton fibers were placed at all face mask edges to check whether they moved when participants were asked to take a deep breath; and third, a qualitative fit test was conducted using the FT-30 Qualitative Fit Test Kit, bitter version (3 M). Only data from patients with a properly sized mask that passed the 3 phases of face mask airtightness testing were included in the study. All subjects were asked to wear face mask continuously except for when eating or drinking. Smokers were excluded since cigarette smoking is known to have deteriorating effects on the ocular surface^19^. Furthermore, subjects were invited not to use video terminal for more than 2 h during the study period in order to avoid a further recognized risk factor for ocular surface impairment ^20^.

### Statistical analysis

Statistical analysis was conducted using R (version 4.0.0) and RStudio (version 1.2.5042) software. Continuous variables are expressed as mean ± standard deviation. A repeated measures ANOVA was used to compare the ocular surface parameters before and after the continuous use of the face mask. A *P* value of less than 0.05 was considered statistically significant.

## Results

A total of 20 healthy subjects (10 males and 10 females, mean age of 25.1 ± 3.9 years) were included in this study. All subjects wore face mask adequately and continuously for 8 h so their data were ultimately used for the analysis. At baseline, the mean values of all objective ocular surface parameters were within the normal range: in particular, mean value of TMH was 0.29 ± 0.07 mm (mm), NIBUT was 11.2 ± 6.4 s, redness score was 0.80 ± 0.28, and meibomian gland dropout was 4.5 ± 4.4%. After 8 h of continuous face mask wearing, mean value of TMH decreased significantly to 0.23 ± 0.07 mm (F [1, 19] = 3.6; *P* < 0.001); conversely, the remaining parameters did not change significantly from T0 to T1 (Table 1).

**Table 1:** Noninvasive ocular surface parameters obtained with Keratograph 5 M in healthy subjects before (T0) and after 8 h of continuous face mask wearing (T1).

Parameter	T0	T1	*P*
Tear meniscus height (mm)	0.29 ± 0.07	0.23 ± 0.07	** < 0.001**
Non-invasive break-up time (s)	11.2 ± 6.4	10.8 ± 7.0	0.833
Redness score	0.80 ± 0.28	0.78 ± 0.38	0.694
Meibomian gland dropout (%)	4.5 ± 4.4	4.4 ± 3.6	0.922

*mm* millimetres; *s* seconds.

Bold values denote statistical significance at the *P* < 0.05 level.

Concerning ocular discomfort symptoms, mean value of OSDI score that fell in the normal range at baseline worsened significantly after 8 h of continuous face mask wearing (from 12.9 ± 12.6 to 19.4 ± 12.0; F [1, 19] = 7.3; *P* = 0.014).

## Discussion

This study evaluated for the first time the diurnal changes of noninvasive parameters of the ocular surface and discomfort symptoms in young healthy individuals who wore continuously face mask for 8 h while working/studying in the same environment. Face mask use was associated with a significant worsening of subjective symptoms; moreover, TMH significantly decreased at T1, possibly because of the increased evaporation of the tear film due to the possible leakage of exhaled air towards the ocular surface. However, albeit statistically significant, the amount of the mean change of TMH was limited, and the parameter remained within the lower range of normality also after continuous use of face mask. On the other hand, NIBUT, ocular redness and meibomian gland dropout did not significantly change over the day.

A previous study correlated the characteristics of face mask wearing in terms of duration with ocular surface signs and symptoms^10^. The authors concluded that the regular daily use of face harmed the ocular surface in the presence of dry eye, inducing a significant worsening of several clinical and molecular parameters when the use is prolonged during the day. On the contrary, the detrimental effects of face mask were more limited in the presence of a healthy ocular surface, even though they tended to become significant when the number of daily hours increased.

Compared to other published studies^7^, our cohort comprised young and healthy subjects with no dry eye disease nor risk factors for its onset, such as contact lens use or previous ocular surgery. This might explain the lesser effect of face mask use upon ocular surface parameters, and in particular upon tear film stability. It is possible to hypothesize that in healthy subjects the tear film homeostatic mechanisms may be capable to counteract the detrimental effects of face mask, at least in the short term. A recent study evaluated healthy subjects by means of invasive tests on 3 separate days: before wearing face mask for more than 30 min (initial examination); after wearing a mask for 8 h; after 15 days of face mask wearing with specific instruction (at least 8 h a day with the open portion of the mask facing the ocular surface taped down from the nostrils to the zygomatic bone with flaster tape)^9^. Face mask use was associated with a statistically significant worsening of all parameters, including OSDI score. More recently, another study reported a significant worsening of discomfort symptoms and break-up time following work-day use of face mask^11^. However, the study enrolled healthcare professionals who exhibited ocular surface parameters outside thermal range already at baseline. It is difficult to compare our results with those from the above-mentioned studies due to differences in time points, types of workout and populations. Nevertheless, symptoms worsened after face mask wearing in all studies regardless of study design and patients’ characteristics, confirming the deleterious effects in terms of ocular discomfort.

Although our study is original, it is affected by some limitations that deserve mentioning. Firstly, the limited sample size could have hampered the detection of statistical significance for some parameters. Secondly, implementing our noninvasive workout with other tests (e.g. corneal fluorescein staining and conjunctival lissamine green staining) could have provided further insights in the onset of this condition. However, all the subjects included were volunteers enrolled in the University while working or studying, so we decided to avoid the use of invasive tests according to the study protocol. Thirdly, we did not evaluate the effect of the taping of the upper mask edge. This strategy has been associated with improved ocular surface stability and discomfort symptoms^8,9^. Nevertheless, this maneuver is not always reproducible and it can induce irritation in the skin and/or disturb the normal eye lid dynamics up to cause ectropion, thus introducing the risk of a possible bias in the evaluation of ocular surface parameters^9^. Finally, diurnal variation of tear meniscus volume has been reported in the past by strip meniscometry self-examination in healthy individuals, with highest values recorded upon awakening that gradually decreased in the evening^21^. Therefore, including and longitudinally examining a control group of individuals who do not wear face mask (to date not yet feasible in a medical facility) could provide further insights in daily variation of ocular surface parameters.

In conclusion, face mask use in young healthy subjects led to decreased TMH associated with the onset of ocular discomfort symptoms. Nevertheless, TMH remained within the normal range also at T1 and the remaining parameters—in particular NIBUT—were not significantly affected by face mask use, possibly reflecting the compensatory effect of the ocular surface system that was able to maintain its homeostasis in young healthy individuals.

## Data Availability

The datasets used and/or analysed during the current study available from the corresponding author on reasonable request.
